# Comprehensive biochemical and radiological assessment of clinical characterization pertaining to bilateral adrenal masses: a multi-center cross-sectional investigation

**DOI:** 10.1007/s12020-026-04667-8

**Published:** 2026-05-29

**Authors:** Gamze Akkus, İrem Kolsuz, Yusuf Kemal Arslan, Ferhatcan Pişkin, Mehtap Evran, Murat Sert, Tamer Tetiker, Yahya Erdem İnce, Ali Örs, Nusret Yılmaz, Özgür Ceylan, Berna Öğmen, Beril Turan Erdoğan, Cevdet Aydın, Bekir Çakır, Özlem Üstay, Yağmur Göksoy Solak, Güzin Fidan Yaylalı, Ayten Eraydın, Semin Melahat Fenkçi, Şenay Topsakal, Yusuf Öztürk, Muhammet Kocabaş, Mehmet Sözen, Berrin Çetinarslan, Zeynep Cantürk, Alev Selek, Emre Gezer, Fatma Öktem, Merve Şimşek Dilli, Fettah Acıbucu, Süheyla Görar, Hüseyin Yağcı, Püren Gökbulut, M. Eda Ertörer, Gülay Şimşek Bağır, Ziynet Alphan Üç, Emre Sedar Saygılı, Büşra Tunç Topuz, Mutlu Güneş, Seher Çetinkaya Altuntaş, Elif Güneş, Lezzan Keskin, Kader Uğur Aksoy

**Affiliations:** 1 Present Address: Faculty of Medicine, Division of Endocrinology, Cukurova University, Adana, Türkiye; 2 Faculty of Medicine, Department of Biostatistics, Cukurova University, Adana, Türkiye; 3 Faculty of Medicine, Department of Internal Medicine, Department of Radiology, Çukurova University, Adana, Türkiye; 4 Faculty of Medicine, Division of Endocrinology, Akdeniz University, Antalya, Türkiye; 5 Faculty of Medicine, Endocrinology and Metabolism Diseases Department, Yıldırım Beyazıt University, Ankara, Türkiye; 6 Bilkent City Hospital, Endocrinology and Metabolism Diseases Department, Ankara, Türkiye; 7 Faculty of Medicine, Division of Endocrinology, Marmara University, İstanbul, Türkiye; 8 Department of Endocrinology and Metabolism, Faculty of Medicine, Pamukkale University, Denizli, Türkiye; 9 Department of Endocrinology and Metabolism, Necmettin Erbakan University, Meram School of Medicine, Konya, Türkiye; 10 Department of Endocrinology and Metabolism, Necmettin Erbakan University, Meram School of Medicine, Necmettin Erbakan University, Konya , Türkiye; 11 Department of Endocrinology and Metabolism, Kocaeli University Faculty of Medicine, Kocaeli, Türkiye; 12 Division of Endocrinology, Department of Internal Medicine, Adana Health Practice and Research Center, University of Health Sciences, Adana, Türkiye; 13 Department of Endocrinology and Metabolism, University of Health Sciences, Antalya Training and Research Hospital, Antalya, Türkiye; 14 Department of Endocrinology and Metabolism, Ankara Training and Research Hospital, University of Health Sciences, Ankara, Türkiye; 15 Faculty of Medicine, Division of Endocrinology and Metabolism, Baskent University, Adana , Türkiye; 16 Department of Translational Medicine, Institute of Health Sciences, Cukurova University, Adana, Türkiye; 17 Department of Endocrinology and Metabolism, Usak Training and Research Hospital, Usak, Türkiye; 18 Division of Endocrinology and Metabolism, Faculty of Medicine, Canakkale Onsekiz Mart University, Canakkale, Türkiye; 19 Department of Endocrinology, Metabolism and Diabetes, Health Sciences University, Bursa Yuksek Ihtisas Training and Research Hospital, Bursa , Türkiye; 20 Department of Endocrinology, Metabolism and Diabetes, Bursa State Hospital, Health Sciences University, Bursa, Türkiye; 21 Department of Endocrinology, Faculty of Medicine, Turgut Özal University, Malatya, Türkiye; 22 Department of Internal Medicine, Department of Endocrinology, Dr. Firat University, Elazig, Türkiye

**Keywords:** Bilateral adrenal masses, Autonomous cortisol secretion, Cushing’s syndrome, Primary aldosteronism, Hounsfield Units, Computed tomography

## Abstract

**Background:**

Bilateral adrenal masses are increasingly diagnosed in routine clinical practice due to widespread use of radiological assessments. Although bilateral adrenal masses consist of heterogenous etiologies including hyperplasia and infiltrative lesions, a majority of them are adrenocortical adrenal lesions which are pathologically confirmed as adenomas. We aim to elucidate the clinical, biochemical, and radiological attributes in individuals exhibiting bilateral adrenal masses, utilizing an extensive sample cohort derived from various medical centers across our nation.

**Methods:**

Retrospective analyses of 1250 patients (female vs. male, 774 vs. 476) with adrenal masses presenting from seventeen endocrine centers between 2010 and 2025 in Turkiye were carried out. Patients presenting with adrenal masses underwent comprehensive clinical, hormonal, and radiological evaluations. Data were obtained from centers located in different provinces across Turkey as part of the study, and a total of 1180 patients were included in the study. After the initial work-up hormonal assessment, the radiological evaluation of adrenal gland neoplasms was conducted employing both non-contrast and contrast-enhanced computed tomography (CT), as well as magnetic resonance imaging (MRI). All imaging acquisitions adhered to established protocols, and the analyses were executed by radiologists with expertise in abdominal imaging. In non-contrast computed tomography (CT) imaging, attenuation values of lesions were quantified in Hounsfield Units (HU), with lesions exhibiting values of ≤ 10 HU classified as indicative of lipid-rich adenoma.

**Results:**

Mean age of all patients was 59.6 ± 10.7 and 735 patients (62.3%) had nonfunctioning adenoma (NF), 235 (19.9%) had autonomous cortisol secretion (ACS), 78 (6.6%) Cushing’s syndrome (CS), and 132 (11.2%) were diagnosed with primary aldosteronism (PA) in the clinical diagnosis distribution. When clinical diagnosis groups (NF, ACS, CS, PA) were compared in terms of biochemical parameters, basal cortisol levels differed between the groups (*p* = 0.033). Furthermore, these levels were higher in the CS group than in the PA and NF groups (*p* = 0.015 and *p* = 0.022, respectively). ACTH, circadian cortisol at 23:00, and cortisol levels after 1 mg and 2 mg dexamethasone differed between the groups (*p* < 0.001). ACTH levels were found to be lower in ACS and CS compared to NF and PA (*p* < 0.001). The most common radiological diagnosis was adenoma (*n* = 1180, 92.3%). Radiological evaluation was available in 829 cases (74.0%), and the HU value in the right adrenal lesions was below 10 in 84.6% of the cases and was homogeneous in 92.2%. In the left adrenal lesions, the HU value was below 10 in 88% of the cases and 92.6% were assessed as homogeneous. MRI was available in 492 cases (41.3%), and 89% of the lesions evaluated in the dual echo sequence were consistent with fat-rich adenoma. A significant correlation between the clinical diagnosis and HU of adrenal masses (right and left sided) was not observed (*p* = 0.244, 0.215). Mean adenoma size of the right (20.7 ± 13.3 vs. 19.9 ± 12.5) and the left adenoma was similar.

**Conclusion:**

In the present study, a significant majority of the subjects were recognized as possessing non-functional adenomas, whereas the other participants were categorized as demonstrating ACS, CS, and PA during the clinical evaluation, respectively. A significant proportion of the patients displayed similar radiological properties concerning HU, defined by consistency despite the presence of hormonal attributes.

## Introduction

Adrenal incidentaloma (AI) is described as an adrenal mass detected in adrenal imaging without suspected adrenal disease. The current guidelines recommend evaluating adrenal masses which are larger than 10 millimeters (mm). Enhanced imaging techniques have raised the prevalence of AI to 4.4% in radiological series compared to autopsy data (1-8.7%). Although a majority of AI consists of unilateral tumors, bilateral AI are found in up to 15% of the cases [1]. The incidence of bilateral adrenal insufficiency has been approximated to range between 0.3 and 0.6% within the general population. Bilateral adrenal neoplasms represent a diverse array of underlying etiological factors. The predominant etiologies associated with these neoplasms include adenomas, Cushing’s disease, bilateral macronodular adrenal hyperplasia, adrenocortical carcinoma, congenital adrenal hyperplasia, non-adrenal neoplasms, infiltrative lesions, infectious processes, and adrenal hemorrhage [2]. The traditional diagnostic hormonal evaluation of these neoplasms encompasses the administration of 1 mg dexamethasone suppression test (DST), the measurement of urinary fractionated metanephrines, or the assessment of plasma aldosterone to renin ratio in cases indicative of primary aldosteronism. Furthermore, all neoplasms ought to be subjected to imaging modalities to ascertain whether the adrenal masses manifest as homogeneous and lipid-rich lesions [3]. Recommendation of diagnostic pathway for bilateral masses are also clinical and hormonal assessments identical to that of unilateral adrenal incidentaloma. After the imaging work-up, a 4 different option schema such as, (1) bilateral macronodular hyperplasia, (2) bilateral adrenal adenomas, (3) two morphologically similar, but non-adenoma-like adrenal masses and (4) two morphologically different adrenal masses have been suggested in the current guidelines [4].

Although the distribution of etiologies of bilateral AI is relatively different from unilateral AI, limited studies reported the most common etiologies were metastasis, primary macronodular hyperplasia and bilateral adrenal adenomas. The contextual framework within which an adrenal mass is diagnosed modifies the probability of diverse etiological factors. It is critical to delineate the metastatic lesions associated with adrenal insufficiency. Following the exclusion of the possibility that the lesions are metastatic; bilateral adrenal adenomas typically present an elevated risk of mild autonomous cortisol secretion (MACS), which correlates with the exacerbation of cardiovascular disease outcomes [5]. MACS previously termed as Subclinical Cushing Syndrome is described as slightly elevated cortisol levels when post-DST is higher than 50 nmol/liter (> 1.8 mcg/dl). For patients who cannot suppress cortisol levels after 1 mg DST, further evaluation (2 days 2 mg DST or urinary cortisol levels) has been suggested to identify MACS clinical entity clearly. Although some panelists believe this clinical conundrum is a physiological part of cortisol secretion, several studies show several cardiovascular and metabolic complications in patients with MACS [6]. According to recent autonomous evaluations, the natural progression of the disease has been associated with an elevated mortality rate attributed to autonomous cortisol secretion. In less common cases, both adrenals exhibit significant enlargement because of the presence of multiple nodules larger than 1 cm, referred to as primary macronodular adrenal hyperplasia [7, 8]. The 2022 World Health Organization classification renamed primary bilateral macronodular adrenal hyperplasia as PBMAD and there has been increased incidental diagnosis reported in studies in the past decade. It is unequivocally established that all patients diagnosed with bilateral adenomas are predisposed to the potential development of pheochromocytomas or aldosteronomas, analogous to autonomous cortisol secretion. Therefore, for incidentally discovered bilateral adrenal masses a comprehensive assessment should be initiated and meticulous clinical and biochemical examinations should be conducted for signs and symptoms of hormone excess.

Despite the recent guidelines issued by the European Society of Endocrinology (ESE) and the European Network for the Study of Adrenal Tumors (ENSAT) offering rigorous scientific recommendations concerning bilateral adrenal masses [4], it has been observed that there is a significant lack of empirical research documenting the hormonal evaluation of such masses. In the present investigation, we seek to systematically observe and delineate the clinical, hormonal, and radiological attributes of individuals presenting with bilateral adrenal masses arising from diverse etiological factors. In addition, we discussed bilateral adrenal masses extensively by comparing radiological and biochemical assessments in those patients.

## Materials and methods

### Patient Population

**Fig. 1:**
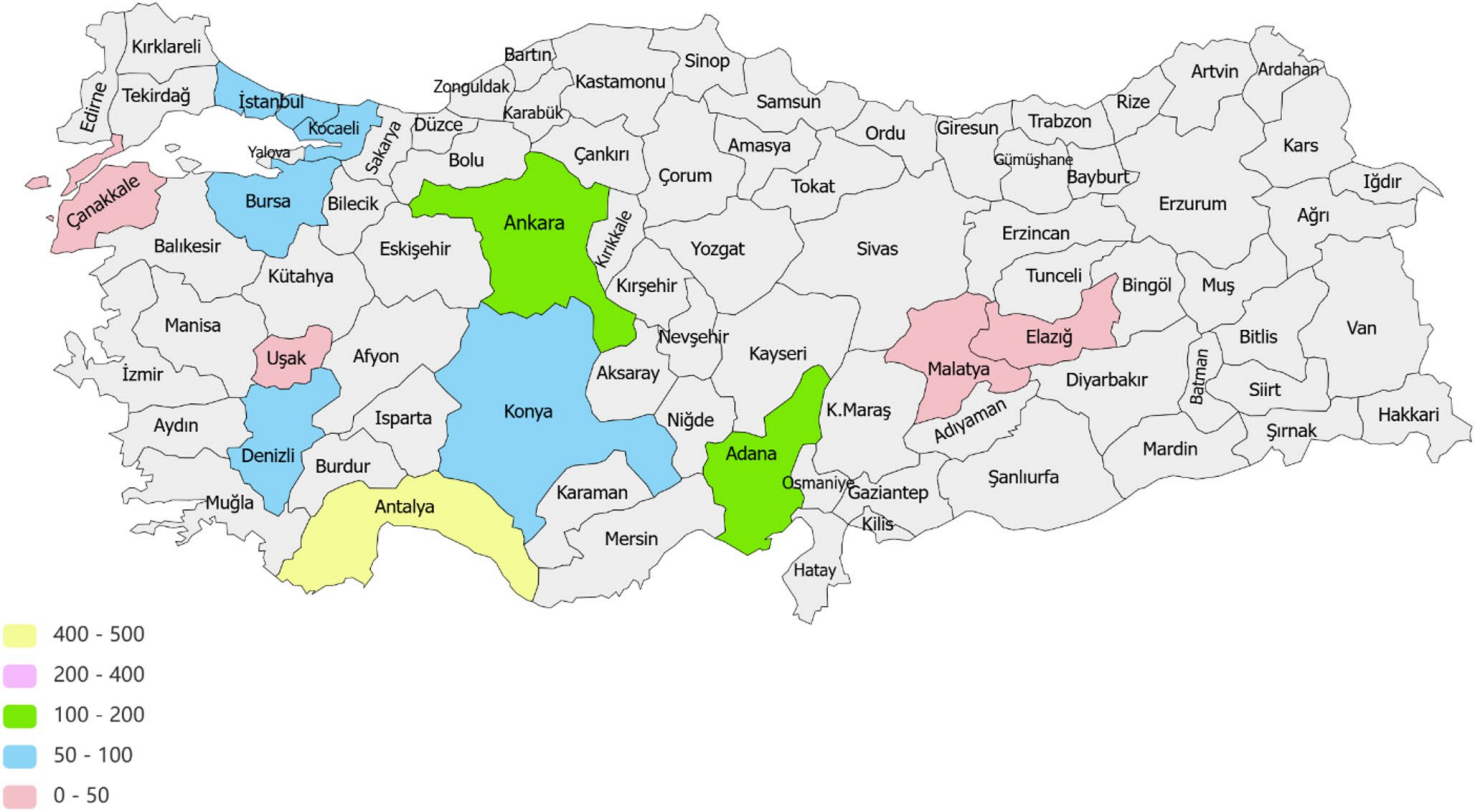
Distribution of the patients included in the study by province

This retrospective multicenter study was approved by the Cukurova University Ethical Committee in 2024 (No:149). Patients exhibiting bilateral adrenal masses as detected through Computed Tomography (CT) or Magnetic Resonance Imaging (MRI) were systematically identified and assessed in accordance with the predetermined inclusion criteria. The inclusion criteria encompassed the presence of bilateral adrenal adenomas and a clinical diagnosis of Nonfunctional Adrenal Incidentalomas (NFAIs), Primary Aldosteronism (PA), Cushing’s Syndrome (CS), MACS, or Pheochromocytomas (Pheo). We methodically excluded individuals with late-onset congenital adrenal hyperplasia or those presenting with metastatic disease concomitant with adrenal insufficiency or adrenal hemorrhage. Patients presenting with adrenal masses underwent comprehensive clinical, hormonal, and radiological evaluations. Data were obtained from centers located in different provinces across Turkey as part of the study, and a total of 1250 patients were enrolled for the study. The number of patients by province was presented using a mapping method (Fig. 1). The mean age of the patients was 59.6 ± 10.7 (20–89), and 774 (62.4%) of the cases were female and 476 (37.6%) were male. Patients were subgrouped with clinical and hormonal assessments, including non-functioning adenoma (NFAI), CS, MACS, Primary Aldosteronism (PA).

### Patient evaluation

The diagnostic criteria for the subjects involved in the study were established in accordance with the prevailing guidelines set forth by the European Society [4];

Non-functional Adrenal Incidentaloma (NFAI) is characterized as adrenal lesions exceeding 1 cm (10 mm) in size that exhibit no hormonal secretion.

Autonomous cortisol secretion (ACS) is fundamentally predicated upon the following criterion: if the post-DST cortisol levels exceeding 1.8 µg/dL,

Mild Autonomous Cortisol Secretion (MACS) is characterized by individuals who exhibit no manifestations or indicators of overt Cushing’s syndrome, alongside a post-dexamethasone serum cortisol level exceeding 50 nmol/L (> 1.8 µg/dL), with no additional categorization dependent on the extent of cortisol nonsuppressibility.

Cushing Syndrome (CS) is diagnosed in the presence of discernible Cushingoid features and normal or higher plasma cortisol levels after the 1 mg and 2 mg DST test, increased levels of 24 h urinary cortisol and increased late-night salivary cortisol. In addition, at least one of the endocrine criteria needed to be met: (1) decreased ACTH levels (< 10 pg/mL), (2) loss of diurnal cortisol rhythm (cortisol at midnight > 7.5 mcg/dl), and (3) decreased ACTH levels response to CRH stimulation test.

Individuals presenting with concurrent hypertension or unexplained hypokalemia were classified as having Primary Aldosteronism (PA); therefore, an elevated Aldosterone (pg/mL) to Renin activity Ratio (ng/ml/h) (ARR > 20) was interpreted as indicative of PA, corroborated by affirmative outcomes from confirmatory assessments such as the Saline infusion test.

Pheochromocytoma (Pheo) was diagnosed as increased (3-fold or higher) plasma-free or urinary fractioned metanephrine measurements of patients with AI.

Hypertension (HT) was defined as systolic blood pressure of > 140 mmHg and diastolic blood pressure of > 90 mmHg under normal circumstances.

Diabetes Mellitus (DM) was defined as a Fasting Plasma Glucose (FPG) level of > 126 mg/dL and HbA1c (National Glycohemoglobin Standardization Program) > 6.5% or using an oral antidiabetic medication.

Hyperlipidemia (HL) was diagnosed as a Low-density Lipoprotein (LDL) cholesterol level of > 140 mg/dL, High-density Lipoprotein (HDL) level of < 40 mg/dL, Triglyceride (TG) level of > 150 mg/dL or using any antihyperlipidemic medication.

### Laboratory and clinical assessment

The initial work-up for bilateral adrenal masses involves the evaluation of hormone parameters. Cortisol excess has been excluded in all patients with 1 mg DST. DST requires taking 1 mg of oral dexamethasone at 23–24 h, then measuring the cortisol level the next morning at 8–9 h; normal levels should be < 50 nmol/L (1.8 mcg/dl). Evaluation of catecholamines excess is performed either by a 24-h urinary collection of metanephrines and fractionated catecholamines. In the presence of arterial hypertension or hypokalemia, it is imperative to assess plasma aldosterone and renin levels after a duration of 15 min in a seated position to facilitate the diagnosis of primary aldosteronism. Furthermore, additional specific laboratory analyses should be conducted in accordance with clinical suspicion, which may include evaluating estrogen levels in cases of gynecomastia and measuring DHEAS, total testosterone, and SHBG in instances of hirsutism or virilization, as well as in cases where there is a suspicion of adrenocortical carcinoma.

### Hormone assay

DHEAS (Dehydroepiandrosterone Sulphate), ACTH, and cortisol values were analyzed by using the enzymatic-labeled chemiluminescent immunometric assay method and chemiluminescence (Beckman DXI 800 auto-analyzer; Beckman Coulter Diagnostics, Fullerton, CA, USA), respectively. The High-performance Liquid Chromatography (HPLC) method was used to analyze urine cortisol and.

metanephrine values.

### Imaging

The radiological assessment of adrenal gland neoplasms was conducted employing both non-contrast and contrast-enhanced computed tomography (CT), as well as magnetic resonance imaging (MRI). All imaging acquisitions adhered to established protocols, and the analyses were executed by radiologists with expertise in abdominal imaging.

In non-contrast computed tomography (CT) imaging, attenuation values of lesions were quantified in Hounsfield Units (HU), with lesions exhibiting values of ≤ 10 HU classified as indicative of lipid-rich adenoma. In contrast-enhanced CT imaging, both absolute and relative contrast washout ratios were derived from images captured during the early (portal venous) and delayed phases; an absolute washout ratio of ≥ 60% or a relative washout ratio of ≥ 40% was deemed compatible with the diagnosis of adenoma.

In the realm of magnetic resonance imaging, both chemical shift (in-phase and opposed-phase) sequences were employed to all subjects. Lesions that manifested a diminution in signal intensity within opposed-phase imaging modalities were categorized as lipid-rich adenomas, whereas lesions that did not exhibit substantial signal attenuation were evaluated for the potential existence of non-adenomatous neoplasms. Furthermore, in T2-weighted sequences, the parameters of signal intensity, homogeneity, and the occurrence of necrosis or hemorrhage were meticulously analyzed.

Radiologically, lesions that were not classified as adenomas were categorized as metastases, pheochromocytomas, or adrenocortical carcinomas, predicated upon characteristics such as elevated attenuation values, insufficient contrast washout, the absence of signal loss in chemical shift sequences, and a heterogeneous architecture. The imaging findings were meticulously assessed in conjunction with clinical, biochemical, and pathological information to facilitate the formulation of a definitive diagnosis.

### Statistical analysis

Categorical measurements were summarized as numbers (n) and percentages (%), while numerical measurements were summarized as mean±standard deviation or median and quartiles (Q1-Q3). The chi-square test statistic was employed to compare non-categorical variables between groups. The Kolmogorov-Smirnov test was used to assess the normality of the numerical measurements. The Student’s t-test was applied to compare age variables that exhibited a normal distribution across gender groups. In clinical diagnosis groups, one-way ANOVA was employed for the general comparison of numerical variables when assumptions were met, and the Kruskal-Wallis test was used when assumptions were not met. In cases where significance was obtained in the Kruskal-Wallis test, the Dunn test was used for pairwise comparisons of groups, and adjusted p-values were presented. In graphical representations, the distribution of patient frequencies in the centers was presented as a map of Turkey, while the percentile values for HU measurements were presented as a percentile plot. The percentile plot was constructed using R (version 4.3.1) and R Studio (version 2023.06.1; Posit PBC, Boston, MA, USA). The statistical analysis of the data was conducted using IBM SPSS 20 (Armonk, NY: IBM Corp.) software. Statistically significant results were defined as those with a p-value less than 0.05.

## Results

### Demographical parameters

**Fig. 2:**
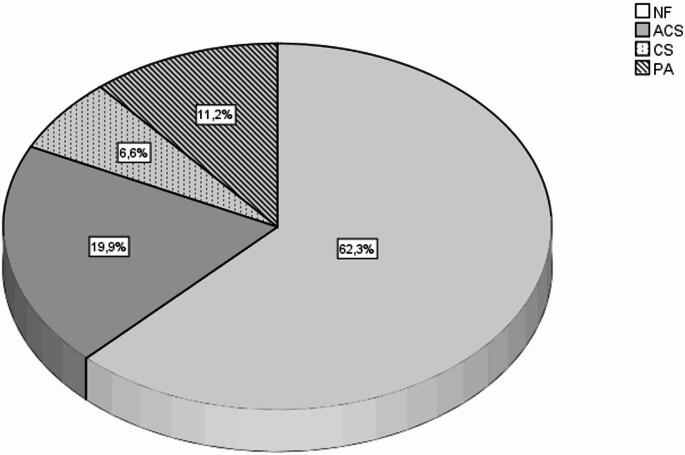
Distribution of of etiologies

When imaging indications were examined, adrenal masses were most frequently detected incidentally (70.0%), with 131 (11.4%) presenting with gastrointestinal symptoms, 86 (7.5%) with secondary hypertension, and 128 (11.1%) with adrenal masses detected during imaging for other reasons. In the clinical diagnosis distribution, 735 patients (62.3%) had nonfunctioning adenoma (NF), 235 (19.9%) had autonomous cortisol secretion (ACS), 78 (6.6%) Cushing’s syndrome (CS), and 132 (11.2%) were diagnosed with primary aldosteronism (PA) Fig. 2. The most common radiological diagnosis was adenoma (*n* = 1180, 92.3%). This was followed by bilateral macronodular adrenal disease (BMAD) in 38 cases (3.5%), metastasis in 16 cases (1.5%), myelolipoma in 9 cases (0.8%), hemorrhage in 3 cases (0.3%), pheochromocytoma in 2 cases (0.2%), and adrenocortical carcinoma in 2 cases (0.2%). In terms of comorbidities, 919 cases (77.0%) had at least one additional disease. When comorbidities were examined, it was seen that the most common was hypertension (60.8%), followed by diabetes mellitus in 470 patients (39.6%) and hyperlipidemia in 417 patients (35.1%). The average duration of DM diagnosis was 10.3 ± 6.2 years, 10.5 ± 5.9 years for HT, and 9.3 ± 5.7 years for HL.

### Hormonal evaluation

In the study, the median basal cortisol value was calculated as 13.4 µg/dl (Q1:10.2–Q3:17.6), ACTH as 12.4 ng/L (7.6–19.4), and circadian cortisol level as 6.2 (4.1–9.1). In the dexamethasone suppression test, cortisol levels were 1.5 (1.1–2.4) after 1 mg and 2.4 (1.8–3.9) after 2 mg. Aldosterone was 14.9 ng/dl (9.3–28.4), plasma renin activity was 1.2 ng/ml/hour (0.4–3.1), and the median aldosterone/renin ratio was 10 (3.8–31.4). Results for DHEA-S, 24-hour urinary catecholamines, metanephrine, normetanephrine, urinary cortisol, HbA1c, total cholesterol, LDL cholesterol, triglycerides, sodium, and potassium are presented in Table 1.

**Table 1:** Hormonal, biochemical, metabolic, radiological findings and lesion characteristics

Basal Cortisol (µg/dl)		*n*(%) or Median(Q1-Q3)
		13.4(10.2–17.6)
ACTH (ng/L)		12.4(7.6–19.4)
Night-time (23:00) Cortisol		6.2(4.1–9.1)
Post-1 mg Dexamethasone Cortisol		1.5(1.1–2.4)
Post-2 mg Dexamethasone Cortisol		2.4(1.8–3.9)
Aldosterone (ng/dl)		14.9(9.3–28.4)
Plasma Renin Activity (ng/ml/hour)		1.2(0.4–3.1)
Aldosterone-to-Renin Ratio (ARR)		10(3.8–31.4)
DHEA-S (µg/dl)		61(35.8-104.8)
24-hour Urinary Catecholamines		80(60–99)
Metanephrine (µg/24 h)		72.7(30.3–129)
Normetanephrine (µg/24 h)		171(60.2-297.5)
24-hour Urinary Cortisol (nmol/24 h)		77(33-171.9)
FPG		99(89–116)
HbA1c		6(5.6–6.9)
Total Cholesterol		199(170–225)
LDL Cholesterol		120(95–143)
Triglycerides		135(100–188)
Sodium (Na)		140(138–142)
Potassium (K)		4.4(4.1–4.7)
CT	No	292(26.0)
	Yes	829(74.0)
Right HU	≥ 10	100(15.4)
	< 10	549(84.6)
Right Heterogeneity	Homogeneous	652(92.2)
	Heterogeneous	55(7.8)
Left HU	≥ 10	77(12.0)
	< 10	566(88.0)
Left Heterogeneity	Homogeneous	646(92.6)
	Heterogeneous	52(7.4)
MRI	No	700(58.7)
	Yes	492(41.3)
Dual-echo sequence lipid-rich adenoma	No	37(11.0)
	Yes	299(89.0)
CT	Right	18(12–26)
	Left	18(11–25)
MRI	Right	20(13–28)
	Left	18(12–25)

ACTH: Adrenocorticotropic hormone, 17-OHP: 17-Hydroxyprogesterone, DHEA-S: Dehydroepiandrosterone sulfate, FPG: fasting plasma glucose, CT: computed tomography, HU: Hounsfield unit, MRI: magnetic resonance imaging

### Radiological and pathological evaluation

Radiological evaluation was available in 829 cases (74.0%), and the HU value in the right adrenal lesions was below 10 in 84.6% of the cases and was homogeneous in 92.2%. In the left adrenal lesions, the HU value was below 10 in 88% of the cases and 92.6% were assessed as homogeneous. MRI was available in 492 cases (41.3%), and 89% of the lesions evaluated in the dual echo sequence were consistent with fat-rich adenoma. On CT, the median lesion size was 18 mm [12–26] on the right and 18 mm [11–25] on the left. MRI measurements were 20 mm (13–28) on the right and 18 mm [12–25] on the left. (Table 1).

A pathological diagnosis was established in a cumulative total of 96 cases, delineated as follows: 56 instances were classified as adenoma, 7 instances as carcinoma, 23 instances as hyperplasia, 2 instances as myelolipoma, and 8 instances as other types.

The comparative analysis of patients exhibiting Nonfunctional (NF), Autonomous Cortisol Secretion (ACS), Cushing’s Syndrome (CS), and Primary Aldosteronism (PA) encompasses an evaluation of hormonal and radiological attributes.

In the comparison between the subgroups, age distributions were similar (*p* = 0.213), but gender distribution differed (*p* = 0.011). The male ratio was 277 (37.7%) in the nonfunctional group, 79 (33.6%) in ACS, 22 (28.2%) in CS, and 64 (48.5%) in PA. No difference was found between the groups in terms of the presence of additional diseases (*p* = 0.285). The rate of additional diseases was 75.1% in the non-functional group, 80% in ACS, 80.8% in CS, and 79.5% in PA. The prevalence of diabetes mellitus differed between the groups (*p* < 0.001), with DM rates of 35.6% in the non-functional group, 41.5% in ACS, 53.2% in CS, and 51.5% in PA. The prevalence of hypertension and hyperlipidemia also differed between the groups (*p* < 0.001 and *p* = 0.002, respectively) (Table 2).

**Table 2:** Baseline, biochemical, metabolic, radiological characteristics and imaging findings according to clinical diagnosis

		Clinical Diagnosis				*p*
		NF	ACS	CS	PA	
Gender	Male	277(37.7)	79(33.6)	22(28.2)	64(48.5)	**0.011**
	Female	458(62.3)	156(66.4)	56(71.8)	68(51.5)	
Comorbidity	No	183(24.9)	47(20)	15(19.2)	27(20.5)	0.285
	Yes	552(75.1)	188(80)	63(80.8)	105(79.5)	
DM	No	471(64.4)	137(58.5)	36(46.8)	64(48.5)	**< 0.001**
	Yes	260(35.6)	97(41.5)	41(53.2)	68(51.5)	
HT	No	337(45.9)	87(37)	32(41)	7(5.3)	**< 0.001**
	Yes	398(54.1)	148(63)	46(59)	125(94.7)	
HL	No	503(68.8)	141(60)	47(60.3)	72(54.5)	**0.002**
	Yes	228(31.2)	94(40)	31(39.7)	60(45.5)	
Basal Cortisol (µg/dl)		13.1(10.07–17.6)	13.9(11-17.7)	15.3(10.34–19.7)	12.6(9.53-17)	**0.033**
ACTH (ng/L)		14.9(9.81-22)	7.61(5.35–12.3)	7.52(4–17)	14(10.9–22.9)	**< 0.001**
Night-time (23:00) Cortisol		4.51(3.34–6.8)	6.09(4.55-8)	9.7(7.64–15.7)	5.57(4.14–7.1)	**< 0.001**
Post-1 mg Dexamethasone Cortisol		1.27(0.95–1.65)	2.8(2.19–3.9)	5.15(2.745–11.7)	1.33(0.98–1.84)	**< 0.001**
Post-2 mg Dexamethasone Cortisol		1.69(1.2–2.4)	2.47(1.97–3.71)	4.23(2.7–8.66)	2.7(1.68–5.88)	**< 0.001**
Aldosterone (ng/dl)		14.17(8.89–27.07)	16(9.74–35.6)	12.11(6.1–17.9)	18.55(12.6–28.1)	**< 0.001**
Plasma Renin Activity (ng/ml/hour)		1.515(0.635–3.95)	1.36(0.54–3.6)	1.2(0.61–2.72)	0.165(0.001–0.46)	**< 0.001**
Aldosterone-to-Renin Ratio (ARR)		7.6(3.2–17.6)	8.2(3.3–23)	8.5(3.3–21)	124.5(37.6–7940)	**< 0.001**
DHEA-S (µg/dl)		70.3(43.05-117.88)	44.79(26.1–69.9)	61(19.1–92)	82.8(50–120)	**< 0.001**
24-hour Urinary Catecholamines		80(60-89.5)	80(60–107)	70(50.5–95.5)	80(80–80)	0.622
Metanephrine (µg/24 h)		76.1(36.3–137)	79.43(31.76-133.88)	81.5(43.01–126.5)	25.55(12.7–75.5)	**< 0.001**
Normetanephrine (µg/24 h)		180(84.95–310)	187(66.88-304.31)	206.12(120–318)	44.77(24.78–179.9)	**< 0.001**
24-hour Urinary Cortisol (nmol/24 h)		98.5(40–173)	47.85(24.72–114)	177(80–354)	71.5(26–134)	**< 0.001**
FPG		98(89–115)	98(89–110)	103(90–128)	101(91–121)	0.103
HbA1c		5.9(5.6–6.7)	6(5.7–6.6)	6.4(5.7–7.5)	6(5.6–7.1)	**0.032**
Total Cholesterol		200(173–226)	194(169–224)	204.5(180–230)	188.5(157–214)	**0.032**
LDL Cholesterol		120(96.5-142.5)	121(90–143)	119(101.2–145)	118(87-142.3)	0.543
Triglycerides		134(99–182)	137(100–185)	131.5(108.5-198.5)	136(106–211)	0.345
Sodium (Na)		140(138–142)	140(138–141)	140(138–142)	140(138–142)	0.933
Potassium (K)		4.4(4.1–4.7)	4.4(4.2–4.7)	4.3(4-4.6)	4.2(3.6–4.6)	**< 0.001**
Right HU	< 10	334(88.4)	131(91)	30(78.9)	80(87)	0.244
	10–20	27(7.1)	9(6.3)	3(7.9)	6(6.5)	
	20	17(4.5)	4(2.8)	5(13.2)	6(6.5)	
Right Heterogeneity	Homogeneous	377(91.3)	133(93.7)	42(93.3)	96(96)	0.393
	Heterogeneous	36(8.7)	9(6.3)	3(6.7)	4(4)	
Left HU	< 10	341(89.5)	126(89.4)	33(84.6)	76(89.4)	0.215
	10–20	24(6.3)	8(5.7)	1(2.6)	7(8.2)	
	20	16(4.2)	7(5)	5(12.8)	2(2.4)	
Left Heterogeneity	Homogeneous	378(92)	129(94.2)	43(95.6)	91(92.9)	0.727
	Heterogeneous	33(8)	8(5.8)	2(4.4)	7(7.1)	
Radiological diagnosis	Adenoma	649(91.5)	208(92)	62(86.1)	112(87.5)	-
	BMAD	17(2.4)	15(6.6)	7(9.7)	3(2.3)	
	ACC	0(0)	0(0)	1(1.4)	1(0.8)	
	Metastasis	18(2.5)	1(0.4)	0(0)	0(0)	
	Myelolipoma	10(1.4)	1(0.4)	0(0)	3(2.3)	
	Hemorrhage	3(0.4)	0(0)	0(0)	0(0)	
	Lymphoma	1(0.1)	0(0)	0(0)	0(0)	
	Other	10(1.4)	1(0.4)	2(2.8)	8(6.3)	
CT	Right	17(12–25)	23(15–30)	27(18.5–32.5)	12(8-16.5)	**< 0.001**
	Left	17(12–24)	22(15–30)	23.5(14.5–38)	12(7–18)	**< 0.001**
MRI	Right	17(11–25)	25(16–31)	28(20–38)	17(10–22)	**< 0.001**
	Left	16.5(12–22)	22(15–28)	26(14–40)	17(12–29)	**< 0.001**

Results were presented as n(%) or Median (Q1-Q3). NF: nonfunctioning adenoma, ACS: autonomous cortisol secretion, CS: Cushing syndrome, PA: primary aldosteronism, DM: Diabetes Mellitus, HT: Hypertension, HL: Hyperlipidemia, ACTH: Adrenocorticotropic hormone, 17-OHP: 17-Hydroxyprogesterone, DHEA-S: Dehydroepiandrosterone sulfate, FPG: fasting plasma glucose, HU: Hounsfield unit, BMAD: Bilateral macronodular adrenal disease, ACC: Adrenocortical carcinoma, CT: computed tomography, MRI: magnetic resonance imaging

When clinical diagnosis of the groups (NF, ACS, CS, PA) was compared in terms of biochemical parameters, basal cortisol levels differed between the groups (*p* = 0.033). Furthermore, these levels were higher in the CS group than in the PA and NF groups (*p* = 0.015 and *p* = 0.022, respectively). ACTH, circadian cortisol at 23:00, and cortisol levels after 1 mg and 2 mg dexamethasone differed between the groups (*p* < 0.001). ACTH levels were found to be lower in ACS and CS compared to NF and PA (*p* < 0.001). Additionally, circadian cortisol levels were observed to be higher in CS compared to the other groups. Furthermore, the nighttime cortisol levels in the ACS group exhibited a higher mean compared to the NF group (adjusted *p* = 0.040). Furthermore, a substantial variation was observed among the clinical diagnosis groups with respect to aldosterone, renin activity, and ARR. The renin activity in the PA group was suppressed, and the ARR in this group was significantly higher compared to the other groups (all adjusted *p* < 0.001 for PA). Levels of DHEA-S were found to be lower in ACS and higher in PA (*p* < 0.001 for ACS–NF and *p* < 0.001 for ACS–PA). Levels of metanephrine and normetanephrine were found to be lower in patients with PA (*p* < 0.001). A significant difference was observed in the 24-hour urinary cortisol levels between the two groups; cortisol levels were lower in the ACS group and higher in the CS group (*p* < 0.001). Furthermore, statistically significant differences were observed between the groups for HbA1c and total cholesterol. A comparison of potassium levels across the three groups revealed that the PA group exhibited lower levels in comparison to the NF and ACS groups (*p* < 0.001 and *p* < 0.001, respectively) (Table 2).

**Fig. 3:**
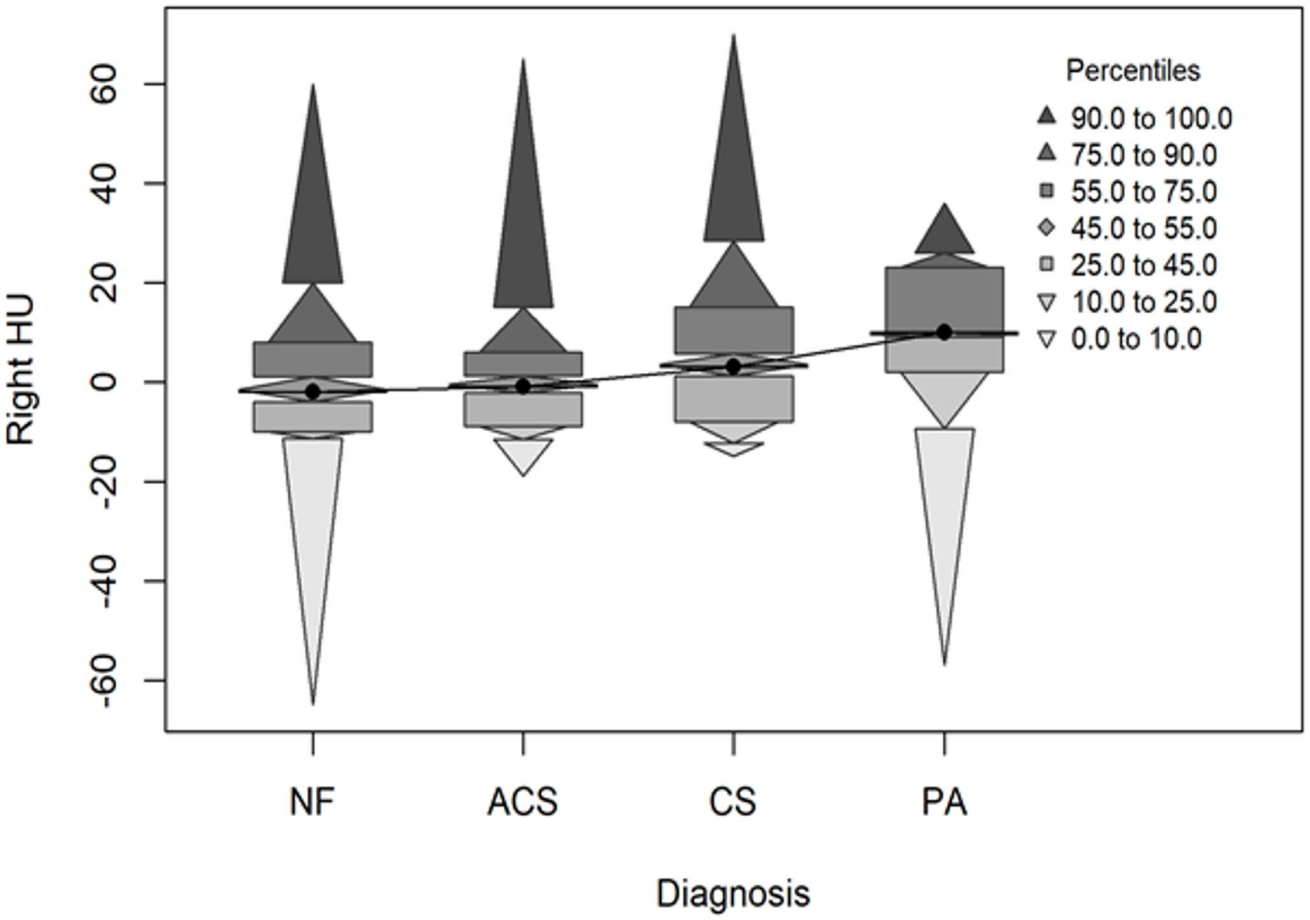
Distribution of right HU values across clinical diagnosis groups (percentile plot)

Table 2 shows that differences were observed between the groups only in terms of lesion size. In CT measurements, both the right and the left lesion sizes varied according to the clinical diagnosis of the groups (*p* < 0.001 for both right and left); the median size was higher in the ACS and especially the CS group, and lower in the PA group. Similarly, MRI showed differences in the right and the left sizes between the groups (both *p* < 0.001). Lesions were larger in the CS and ACS groups and smaller in the PA group. A statistically significant difference was found in the right adrenal HU distribution between the clinical diagnosis of the groups (*p* = 0.040), and pairwise comparisons showed that the PA group had higher right HU values than the non-functioning and ACS groups (Fig. 3). The left adrenal HU distribution was similar in the diagnosis of the groups (*p* = 0.092).

**Fig. 4:**
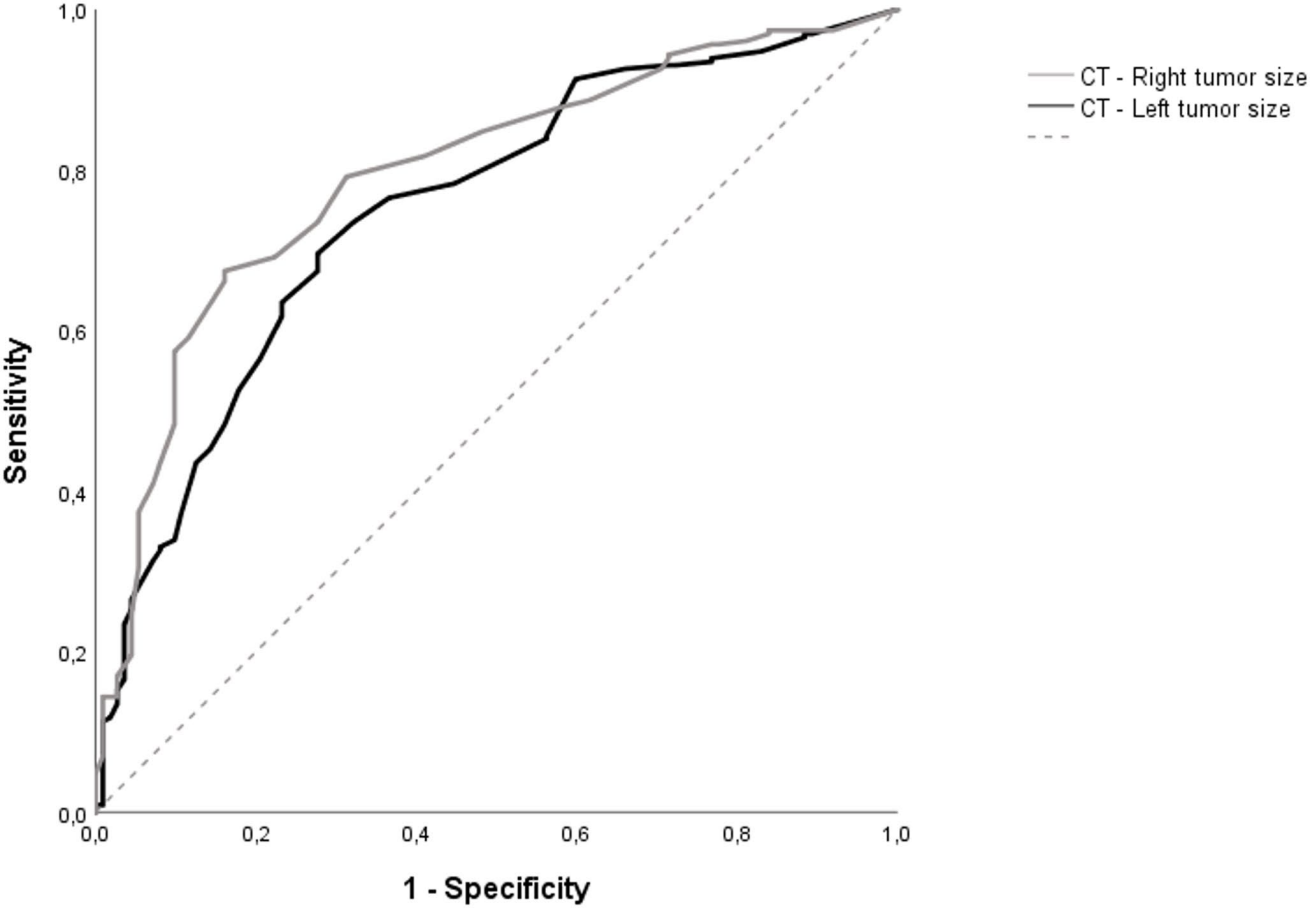
ROC curve for determining the diagnostic role of CT tumor size in differentiating the PA group from the ACS/CS groups

ROC curve analysis was used to determine whether adrenal nodule size could statistically significantly distinguish ACS/CS from PA, and the area under the curve was found to be significant for both sides (*p* < 0.001). For right-sided lesions, the cutoff value for adrenal nodule size was determined to be > 18.5 mm. At this value, the area under the curve (AUC) was 0.795 (95% CI: 0.746–0.845), with a sensitivity of 67.4% and a specificity of 83.9%. Similarly, for left-sided lesions, an optimal cutoff value of > 16.5 mm yielded an AUC of 0.752 (95% CI: 0.698–0.807) with a sensitivity of 69.6% and a specificity of 72.3% (Fig. 4). A multinomial logistic regression analysis was conducted to compare hormonal subtypes with the NF group. This analysis identified the size of adrenal neoplasms as an independent predictive factor across all clinical categories (Table 3). In the multivariate model for ACS, being female (OR for males: 0.642, 95% CI: 0.423–0.974, *p* = 0.037) and having larger tumor size (OR: 1.032, 95% CI: 1.015–1.049, *p* < 0.001) were found to be associated with the diagnosis. Similarly, tumor size was identified as the only independent predictor for CS, with an observed increase in likelihood of CS of 3.9% for each millimeter of size increase (OR: 1.039, 95% CI: 1.014–1.065, *p* = 0.002). While the size of the adrenal masses was found to be significant in the primary aldosteronism group, this was not the case in the other groups. In the primary aldosteronism group, the diameter of the adrenal masses was found to be smaller compared to the non-functioning group (OR: 0.926, 95% CI: 0.898–0.955). While gender was initially associated with PA in univariate analysis (*p* = 0.020), this association was not statistically significant in the multivariate model (*p* = 0.255). In the final model, no association was found between age and Hounsfield Unit values and any functional subtype.

**Table 3:** Multinomial Logistic Regression Analysis for Predicting Hormonal Subtypes (ACS, CS, and PA) Relative to Non-functional (NF) Adrenal Masses

	Univariate	*p*	Multivariable	*p*
	OR (%95 CI)		OR (%95 CI)	
ACS vs. NF				
Age	1.004 (0.990–1.018)	0.606	1.015 (0.996–1.034)	0.132
Sex (Male*)	0.837 (0.615–1.141)	0.260	0.642 (0.423–0.974)	**0.037**
Size of adrenal neoplasms (mm)	1.027 (1.014–1.041)	**< 0.001**	1.032 (1.015–1.049)	**< 0.001**
HU (> 10**)	0.761 (0.426–1.361)	0.357	0.610 (0.330–1.127)	0.115
CS vs. NF				
Age	0.981 (0.960–1.003)	0.083	0.993 (0.962–1.026)	0.675
Sex (Male*)	0.650 (0.388–1.087)	0.101	0.599 (0.284–1.261)	0.177
Size of adrenal neoplasms (mm)	1.034 (1.015–1.054)	**0.001**	1.039 (1.014–1.065)	**0.002**
HU (> 10**)	2.090 (0.959–4.556)	0.064	1.500 (0.657–3.423)	0.336
PA vs. NF				
Age	0.991 (0.974–1.009)	0.324	0.993 (0.972–1.015)	0.553
Sex (Male*)	1.556 (1.072–2.259)	**0.020**	1.314 (0.821–2.106)	0.255
Size of adrenal neoplasms (mm)	0.927 (0.904–0.952)	**< 0.001**	0.926 (0.898–0.955)	**< 0.001**
HU (> 10**)	0.929 (0.484–1.782)	0.824	1.399 (0.699-2.800)	0.343
ACS vs. NF				
Age	1.004 (0.990–1.018)	0.606	1.011 (0.989–1.034)	0.317
Sex (Male*)	0.837 (0.615–1.141)	0.260	0.737 (0.420–0.967)	0.187
Size of adrenal neoplasms (mm)	1.027 (1.014–1.041)	**< 0.001**	1.020 (1.002–1.038)	**0.035**
HU (> 10**)	0.761 (0.426–1.361)	0.357	0.556 (0.282–1.097)	0.091
Potassium	1.082 (0.788–1.487)	0.626	1.037 (0.640–1.682)	0.881
Post 1 mg-DST Kortizol	2.192 (1.918–2.506)	**< 0.001**	2.058 (1.719–2.473)	**< 0.001**
CS vs. NF				
Age	0.981 (0.960–1.003)	0.083	1.013 (0.972–1.057)	0.533
Sex (Male*)	0.650 (0.388–1.087)	0.101	0.810 (0.329–1.992)	0.646
Size of adrenal neoplasms (mm)	1.034 (1.015–1.054)	**0.001**	1.029 (0.997–1.061)	0.075
HU (> 10**)	2.090 (0.959–4.556)	0.064	1.350 (0.489–3.731)	0.563
Post 1 mg-DST Kortizol	2.541 (2.203–2.930)	**< 0.001**	2.371 (1.950–2.884)	**< 0.001**
Potassium	0.553 (0.342–0.894)	**0.016**	0.709 (0.278–1.803)	0.470
PA vs. NF				
Age	0.991 (0.974–1.009)	0.324	0.999 (0.975–1.024)	0.949
Sex (Male*)	1.556 (1.072–2.259)	**0.020**	1.284 (0.771–2.138)	0.336
Size of adrenal neoplasms (mm)	0.927 (0.904–0.952)	**< 0.001**	0.931 (0.902–0.961)	**< 0.001**
HU (> 10**)	0.929 (0.484–1.782)	0.824	1.077 (0.502–2.307)	0.850
Post 1 mg-DST Kortizol	1.410 (1.177–1.688)	**< 0.001**	1.516 (1.205–1.906)	**< 0.001**
Potassium	0.261 (0.179–0.380)	**< 0.001**	0.413 (0.252–0.678)	**< 0.001**

*Reference category: Female. **Reference category: HU < 10. NF: Non-functional; ACS: Autonomous Cortisol Secretion; CS: Cushing Syndrome; PA: Primary Aldosteronism; HU: Hounsfield unit; OR: Odds ratio; CI: Confidence interval

## Discussion

In our cohort, we noted a predominance of non-functioning adenomas, accounting for approximately 60%. Furthermore, there exists a considerable prevalence of autonomous cortisol secretion, estimated at around 20%. Additionally, the functional groups exhibited analogous radiological characteristics, characterized by low Hounsfield units and homogeneity. Moreover, there was an observable increase in cardiometabolic comorbidity associated with hormonally active lesions. These findings align with prior research and established clinical guidelines. We studied patients with bilateral adrenal masses in a large sample size from the multicenter. In a cohort of 1180 cases, the preponderance of patients (62.3%) exhibited nonfunctioning adenomas, whereas the remaining cases were classified as ACS (19.9%), CS (6.6%), and PA (11.2%) based on the clinical assessment, respectively. The female gender was notably prevalent across all sub-groups. Hormonal parameters, encompassing baseline cortisol, adrenocorticotropic hormone (ACTH), the 1 mg dexamethasone suppression test (DST), circadian cortisol levels, dehydroepiandrosterone sulfate (DHEA-S), and the aldosterone-to-renin ratio (ARR), exhibited significant variability among the identified subgroups, in alignment with the anticipated outcomes. In accordance with the hormonal assessments, a significant elevation in basal cortisol levels was detected among subjects diagnosed with Cushing’s syndrome (CS), achieving a statistical significance of *p* = 0.003 and a prevalence rate of 18.8%. In instances of concomitant diseases, hypertension was prevalent across all subgroups; however, diabetes mellitus was more frequently observed in individuals diagnosed with Cushing’s syndrome and primary aldosteronism. The most common radiological diagnosis was adenoma (*n* = 994, 90.8%). Radiological assessment was conducted in 829 instances (74.0%), revealing that the HU value for the right adrenal lesions was recorded as below 10 in 84.6% of the cases, with a homogeneity observed in 92.2% of the instances. In the context of the left adrenal lesions, the HU value fell below 10 in 88% of the cases, with 92.6% categorized as homogeneous. Magnetic Resonance Imaging (MRI) was performed in 492 instances (41.3%), where 89% of the lesions examined through the dual echo sequence were determined to be consistent with fat-rich adenomas.

Adrenal incidentaloma is discovered during testing for another condition and should be tested with biochemical or radiological methods. The prevalence of adrenal incidentaloma has been reported to be 1 to 6%. Incidence of unilateral or bilateral adrenal masses increased 10-fold in the past 2 decades, in parallel to the increase in the number of abdominal CT and MRI. The prevalence is higher among older adults in fifth to seventh decades. Studies [9–11] reported that gender distribution was almost equal in men (45%) and women (55%). Population based studies which represented the mode of discovery indications were incidental (85%), cancer staging imaging (7%), symptoms of hormone excess (7%), abdominal mass effect (< 1%), respectively [12–14]. In the present investigation, a cohort comprising 1180 patients exhibiting bilateral masses was observed, with a calculated mean age of 59.6 ± 10.7 years (ranging from 20 to 89 years); of these, 774 individuals (62.4%) were identified as female, while 476 individuals (37.6%) were categorized as male. Upon the analysis of imaging indications, adrenal masses were predominantly identified incidentally (70.0%), with 131 cases (11.4%) exhibiting gastrointestinal manifestations, 86 instances (7.5%) associated with secondary hypertension, and 128 cases (11.1%) where adrenal masses were identified during imaging conducted for alternative clinical considerations. While there may be minor variations in the age of onset, gender distribution, and the rationale for the identification of adrenal masses across different populations, the findings tend to align consistently along a similar framework.

The causes of bilateral adrenal masses vary depending on whether recruitment was from endocrinology clinics or from surgical series where adrenal cancers and secreting tumors are over presented, leading to overestimation of the real prevalence of malignancy. Although many of the studies are generally described and defined as unilateral adrenal masses in nature, recent fewer studies demonstrated the most common etiologies are tumoral including adenomas, pheochromocytoma, hyperplasia, nontumoral lesions such as metastases, lymphoma and infections, amyloidosis and hemorrhage, pathologically [15–17]. It is known that the distribution of etiologies of bilateral masses may differ from unilateral masses. After a detailed hormonal work up for cortisol excess, hyperaldosteronism and pheochromocytoma, two additional investigations should be performed to rule out adrenal insufficiency related with adrenal metastases or late onset congenital adrenal hyperplasia. With the exception of hyperplasia and metastases, nonfunctional adenomas represent the most prevalent category observed. We observed that the most common diagnosis in the radiological and clinical evaluations was diagnosed as adenoma (90.8%) with homogenous features. Conversely, it has been observed that MACS occurs with greater prevalence in individuals presenting with hyperplasia or adenomas as compared to those with unilateral adenomas, which correlates with their functional characteristics. While a predominant proportion of our subjects (*n* = 735, 62.3%) presented with nonfunctioning adenoma, the remaining cases included 235 (19.9%) exhibiting autonomous cortisol secretion (ACS), 78 (6.6%) diagnosed with Cushing’s syndrome (CS), and 132 (11.2%) identified as having primary aldosteronism (PA), respectively. Patients with mild cortisol secretion might carry an increased risk of osteoporotic, metabolic and cardiovascular complications compared to control patients matched for age, gender and BMI. According to hormone parameters, basal cortisol and ACTH levels were the significant factor leading to the occurrence of metabolic complications. We also observed that the circadian cortisol levels in the ACS group exhibited a higher mean compared to the NF group (adjusted *p* = 0.040). Due to the persistence of cortisol secretion in those patients, they were likely to develop cardiovascular events compared to the patients with NFAI during a conservative follow-up. In the year 2014, Di Damalzi et al. [18] conducted a study involving a retrospective cohort of individuals diagnosed with adrenal incidentalomas, revealing that the survival rates pertaining to all-cause mortality were diminished in patients exhibiting abnormal suppression to a 1 mg dexamethasone suppression test (DST). More recent studies [19–21] have shown cortisol to be an independent risk factor for the presence of comorbidity in patients with adrenal adenomas. In the present study, every cohort exhibited supplementary comorbid conditions, and hypertension was uniformly prevalent across all subcategories, albeit with varying frequencies. However, diabetes was more frequently observed in individuals diagnosed with Cushing’s syndrome (CS) and PA as well. When conducting a comparative analysis of basal cortisol levels, it has been observed that there were mild variations among the subjects. Only the cortisol levels within the CS group were significantly elevated in comparison to the other groups. Patients presenting with bilateral adrenal masses accompanied by hypercortisolism pose a significant challenge in clinical management. While bilateral adrenalectomy is regarded as the optimal intervention, it is imperative to consider the associated complications; namely, the necessity for lifelong adrenal hormone replacement therapy and the risks of Addisonian crisis, which may adversely affect patient outcomes. We additionally noted a disparity in the impairment of levels following administration of 1 mg and 2 mg of dexamethasone among the different groups (*p* < 0.001), even when considering PA. And various research investigations have posited that hyperaldosteronism assumes a novel function in the context of metabolic syndrome, prediabetes, or diabetes mellitus when compared with patients exhibiting NFAI or ACS. We declared a majority of the patients with bilateral masses as adenomas including NF, ACS, CS, PA. Taking into account the hormonal parameters alongside additional factors, we have delineated the PA and the CS group as the cohort exhibiting the greatest vulnerability to comorbid conditions.

For patients with bilateral adrenal masses, each adrenal lesion is assessed at the time of the initial detection according to the same imaging protocol for unilateral adrenal masses to establish whether each nodule is benign or malignant. In most cases, bilateral adrenal masses represent benign adrenal bilateral adrenocortical disease: either bilateral adenomas, macronodular hyperplasia or distinct bilateral nodules with normal or atrophic cortex. Some studies confirmed that incidentally detected bilateral adrenal adenoma were generally described as bilateral adrenal adenomas. Some studies argued that the incidence of malignancy appears to be elevated in instances of bilateral nodules. Zhou et al. [22] conducted an investigation involving 18 cases of bilateral adrenal masses and identified six instances of pheochromocytoma, six instances of adenoma, four instances of lymphoma, and two instances of metastatic disease. According to our findings, the predominant diagnosis identified through radiological assessment was adenoma, which was observed in 1180 instances (90.8%). This was succeeded by bilateral macronodular adrenal disease (BMAD) in 38 instances (3.5%), metastasis in 16 instances (1.5%), myelolipoma in 9 instances (0.8%), hemorrhage in 3 instances (0.3%), pheochromocytoma in 2 instances (0.2%), and adrenocortical carcinoma in 2 instances (0.2%). The value of unenhanced CT and chemical shift MRI is well established and if the HU of the masses is < 10, there is no need to further evaluation [23, 24]. In cases where an additional unenhanced computed tomography scan shows adrenal masses exceeding > 10, the exploration of the most appropriate second-line imaging modality is advised. The results of imaging show that 60% of adenomas are lipid rich with unenhanced HU < 10, 25% with HU between 10 and 19, 15% with HU > 20.

In our studied cohort (*n* = 829, 74.0%), the Hounsfield Unit (HU) measurement for the right adrenal lesions exhibited values below 10 in 84.6% of the instances and demonstrated homogeneity in 92.2% of the cases. Concerning the left adrenal lesions, the HU values were recorded as below 10 in 88% of the cases, with 92.6% being classified as homogeneous. Magnetic Resonance Imaging (MRI) was conducted in 492 instances (41.3%), and it was determined that 89% of the lesions assessed through the dual echo sequence were indicative of fat-rich adenomas. Tumor size is an another important factor to determine the risk of malignancy. Median size of a majority nonfunctional adrenal adenomas (90–95%) was 20–30 mm. In a population study of 1287 patients [21], 6% of adrenal tumors < 2 cm and 9% of adrenal tumors between 2 and 4 cm were malignant, as opposed to 34% of adrenal tumors > 4 cm being malignant. We obtained that the median lesion size was 18 mm [12–26] on the right and 18 mm [11–25] on the left. MRI measurements were 20 mm (13–28) on the right and 18 mm [12–25] on the left. Although the dimensions of the masses were correlated with the existing literature, we posited that mass size could serve as a diagnostic criterion to articulate the functional characteristics of the tumor. The median dimension was notably elevated in the ACS and particularly in the CS group, while it was diminished in the PA group. Correspondingly, MRI demonstrated significant disparities in the dimensions of the right and left masses across the groups (both *p* < 0.001). And we obtained tumor size was identified as the only independent predictor for CS. While the size of the adrenal masses was found to be significant in the primary aldosteronism group, this was not the case in the other groups. In the primary aldosteronism group, the diameter of the adrenal masses was found to be smaller compared to the non-functioning group.

Lesions exhibited greater dimensions in the CS and ACS groups, whereas they were smaller in the PA group. The computed tomography (CT) characteristics, specifically Hounsfield units (HU) or homogeneity, failed to provide any discernible indicators for distinguishing the clinical attributes of the masses associated with autonomous cortisol secretion or primary aldosteronism. Research regarding [25] the differentiation between functional and non-functional adrenal adenomas via magnetic resonance imaging (MRI) and computed tomography (CT) has been relatively scarce. Simone et al. [26] indicated that MRI metrics, specifically the signal intensity index (SII), did not exhibit a statistically significant correlation when comparing hypersecreting adenomas (*n* = 18, 2.8 ± 0.5 cm) with non-hypersecreting counterparts (*n* = 15, 3.1 ± 0.9). Furthermore, Yener et al. [27] posited that MRI measurements or Hounsfield Units (HU) could effectively distinguish individuals exhibiting cortisol secretion (*n* = 13) from those with non-functional adrenal incidentalomas (*n* = 68). They further elaborated that the inverse relationship between tumor lipid composition and hypercortisolism was associated with an elevated rate of cortisol production within the tumor. In the course of our investigation, we did not identify any clinically significant correlation between the radiological findings, including homogeneity, Hounsfield Units (HU), and clinical diagnoses. We posited that the radiological characteristics solely reflect the attributes of the mass rather than the assessment of hormonal levels.

### Strength of the study

In the course of our inquiry, we concentrated on bilateral adrenal adenomas in instances exhibiting hormonal and radiological characteristics involving a substantial cohort of patients. It is acknowledged that the etiopathogenesis and hormonal attributes of bilateral adrenal masses remain inadequately understood due to insufficient data. Subsequent to the presentation of clinical and hormonal assessments, we undertook a comparative analysis of patient subgroups based on biochemical and radiological characteristics. This current study signifies the first systematic investigation focusing on the clinical and radiological assessment of bilateral adrenal adenomas.

### Limitations

The current research was formulated as a retrospective analysis; thus, specific data points were not available. Moreover, the multicenter framework introduces substantial heterogeneity, resulting in uncertainties pertaining to the standardization of hormonal assays, inconsistencies in imaging protocols and their interpretations, or variations in diagnostic thresholds across the diverse centers.

## Conclusion

In the current study, we delineated the clinical, radiological, and hormonal attributes of individuals exhibiting bilateral adrenal masses in a cohort of 1180 patients sourced from various medical centers. A predominant proportion of the patients were identified as having non-functional adenomas, while the remaining individuals were classified as exhibiting autonomous cortisol secretion, Cushing’s Syndrome, and primary aldosteronism during the clinical assessment, respectively. The predominant diagnosis identified was adenoma (90.8%) based on the radiological assessment characterized by < 10 HU and homogeneous features. Upon comparison of the biochemical and radiological characteristics of patients with NF, ACS, Cushing’s syndrome (CS), and primary aldosteronism (PA), it was observed that conditions associated with excess cortisol and aldosterone are more likely to precipitate secondary comorbidities. But we did not obtain any correlation between the CT characteristics, specifically Hounsfield units (HU) or homogeneity and clinical diagnosis. Bilateral adrenal masses constitute a prevalent pathological condition necessitating the implementation of comprehensive diagnostic and therapeutic strategies tailored to the specific circumstances of each case, while considering various factors including the potential for malignancy and the status of hormone secretion. Our findings indicate that the distribution of etiological factors exhibits a resemblance to that of unilateral adrenal masses, with a notable prevalence of adenomas characterized by benign radiological characteristics.

## Data Availability

No datasets were generated or analysed during the current study.
